# Scanning Micromirror Calibration Method Based on PSO-LSSVM Algorithm Prediction

**DOI:** 10.3390/mi15121413

**Published:** 2024-11-25

**Authors:** Yan Liu, Xiang Cheng, Tingting Zhang, Yu Xu, Weijia Cai, Fengtian Han

**Affiliations:** 1 School of Ocean Information Engineering, Jimei University, Xiamen 361021, China; ly_liu@jmu.edu.cn; 2 Pen-Tung Sah Institute of Micro-Nano Science and Technology, Xiamen University, Xiamen 361102, China; zhangtt@stu.xmu.edu.cn; 3 School of Aerospace Engineering, Xiamen University, Xiamen 361102, China; 35120231151744@stu.xmu.edu.cn (Y.X.); 34520241151688@stu.xmu.edu.cn (W.C.); 4 Department of Precision Instrument, Tsinghua University, Beijing 100084, China; hanft99@mails.tsinghua.edu.cn

**Keywords:** PSO-LSSVM, MOEMS, scanning micromirror, photodetector

## Abstract

Scanning micromirrors represent a crucial component in micro-opto-electro-mechanical systems (MOEMS), with a broad range of applications across diverse fields. However, in practical applications, several factors inherent to the fabrication process and the surrounding usage environment exert a considerable influence on the accuracy of measurements obtained with the micromirror. Therefore, it is essential to calibrate the scanning micromirror and its measurement system. This paper presents a novel scanning micromirror calibration method based on the prediction of a particle swarm optimization-least squares support vector machine (PSO-LSSVM). The objective is to establish a correspondence between the actual deflection angle of the micromirror and the output of the measurement system employing a regression algorithm, thereby enabling the prediction of the tilt angle of the micromirror. The decision factor ($R^{2}$) for this model at the *x*-axis reaches a value of 0.9947.

## 1. Introduction

A micro-opto-electro-mechanical system (MOEMS) is a controllable micro-optical system comprising micro-optical elements that are capable of converging, diffracting, and reflecting light beams under the action of micro-electronic and micro-mechanical devices [1]. As the fundamental component of MOEMS, scanning micromirrors have been extensively employed in several fields, including imaging systems [2], optical communication [3], biomedicine [4], spectroscopy [5], and other domains. The detection of micromirrors is primarily categorized according to the sensing method and measurement employed. The most common techniques are inductive [6], piezoresistive, capacitive, and optical [7]. However, the accuracy of the measurement is significantly influenced by a number of factors, including drift overtemperature [8], jitter, asymmetrical process production, external interference [9], and other variables. In the context of microsystems, these factors can have a considerable impact on the scanning effect. To maintain optimal scanning performance of MEMS micromirrors, it is essential to monitor their displacement and angular tilt throughout operation. To calibrate the scanning micromirror measurement system, it is first necessary to identify the correspondence between the actual deflection angle of the micromirror and the output value of the measurement system. Itaru Ishikawa et al. presented a novel integrated sensor design that can be integrated into a scanning micromirror with a vertical displacement resolution of up to 20 nm. However, the linear range of the sensor’s tilt measurement is limited to ±2.5° and it can only detect single-axis motion, with an analog output [7].

There is still much to be done to develop effective monitoring techniques for micromirrors. This paper presents a regression analysis of two-dimensional deflected micromirror data using the PSO-LSSVM algorithm, which achieves a highly accurate prediction of the micromirror tilt angle. A scanning micromirror measurement system integrating a four-quadrant photodetector (PD) with a Vertical-Cavity Surface-Emitting Laser (VCSEL) and a high-precision dual-axis goniometer was first constructed [10]. However, to enable the prediction of micromirror tilt angles, the development of a non-linear model of the two-dimensional deflection motion of scanning micromirrors is still required. The Support Vector Machine (SVM), which is based on the principle of structural risk minimization, is a two-class classification model that was proposed by Cortes and Vapnik in 1995 [11]. It is widely used for research in nonlinear, small-sample, and high-dimensional pattern recognition. Moreover, it has a high degree of generalization capability and is capable of obtaining a unique global optimal solution. The Least Squares Support Vector Machine (LSSVM) represents an improvement on the standard SVM [12], offering a significant improvement in solution speed. The PSO-LSSVM algorithm, which integrates the particle swarm optimization [13] algorithm with the LSSVM algorithm to predict the tilt angle of the micromirror, demonstrates enhanced learning ability and greater adaptability to new samples on this basis. A nonlinear model for the two-dimensional motion of the scanning electrothermal micromirror has been established, thus resolving the inherent limitation of susceptibility to external influences in micromirror test results and enabling high-precision prediction of the micromirror tilt angle.

## 2. Structural and Theoretical Analyses of Monitored Micromirror

### 2.1. The Architecture of the Micromirror Monitored Comprises a Four-Quadrant Photodetector

**Figure 1:**
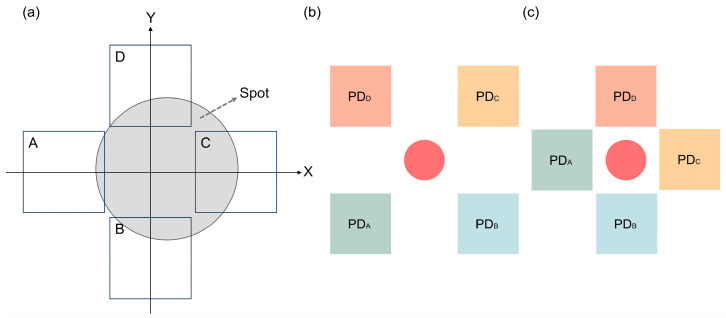
Schematic diagram of the four-quadrant photodetector: (**a**) schematic diagram of a spot in a four-quadrant photodetector; (**b**) QPD_I; (**c**) QPD_II.

The micromirror monitored system comprises a four-quadrant photodetector with a VCSEL integrated in the center, featuring compact. The four-quadrant photodetector receives the light reflected from the micromirror and subsequently converts it into a voltage output. When the micromirror and the photodetectors are in a non-parallel state, the light-receiving area of each photodetector will be different, as shown in Figure 1a. When the photodetectors are illuminated by a reflected spot, they will convert the optical signals into electrical signals and output photocurrents. The magnitude of photocurrent is proportional to the magnitude of the light flux irradiated on each photodetector. However, it is necessary to convert the photocurrents into photo-voltages using operational amplifiers since the values of the photocurrents are generally small.

The four-quadrant photodetector is primarily organized in two distinct arrangements: a square configuration (QPD_I, in Figure 1b) and a cross configuration (QPD_II, in Figure 1c). In practice, the secondary reflection caused by the substrate between the detectors will introduce errors. In QPD_II, the substrate between the detectors is the area near the light source, which has less influence than that of QPD_I. Given the height of the micromirror, it is obvious that there must be a certain vertical distance between the micromirror and the photodetectors to ensure that the micromirror deflects without touching the photodetectors and to allow the necessary space for the chip package and wiring. When the height of the mirror is greater than a certain value, QPD_II exhibits superior linearity and sensitivity. Despite the higher area utilization of QPD_I, the dead zone in QPD_I is smaller. However, given that the activity height of the micromirror is orders of magnitude greater than that of the dead zone, the dead zone and area utilization are no longer significant considerations. Consequently, the QPD_II arrangement offers certain advantages over QPD_I.

**Figure 2:**
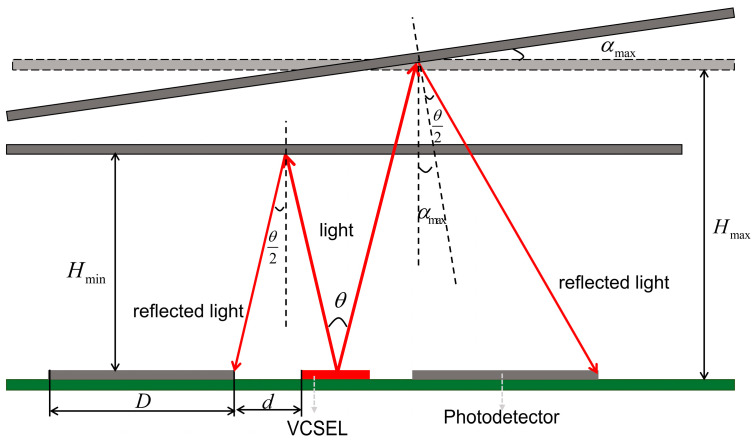
Schematic representation of the relationship of the distance H between the micromirror and the PDs.

For the PD to sense the tilt of the micromirror immediately and still have light intensity at the photodetector when the micromirror reaches the maximum tilt angle, the distance H between the micromirror and the four-quadrant photodetector must be maintained as follows:

(1) $$H_{\min}\geq \frac{d}{2\tan\frac{\theta }{2}}$$

(2) $$H_{\max}\leq \frac{(d+D)\left[\cos(\theta +3\alpha_{\max})+\cos\alpha_{\max}\right]}{\sin(\theta +2\alpha_{\max})\cos\alpha_{\max}}$$

where d is the minimum distance from the VSCEL to the PDs. According to the conditions that H should be satisfied, the beam divergence θ of the VCSEL, and the size of the PD D are mainly taken into account when designing the four-quadrant photodetector, as shown in Figure 2.

The tilt angle measurement of the QPD_II is calculated based on the sum-to-difference ratio of the output voltages of the two coaxial PDs [14]:

(3) $$\alpha_{x}=\frac{U_{x_{1}}-U_{x_{2}}}{U_{x_{1}}+U_{x_{2}}},\alpha_{y}=\frac{U_{y_{1}}-U_{y_{2}}}{U_{y_{1}}+U_{y_{2}}}$$

$U_{x_{1}}$ and $U_{x_{2}}$ represent the output voltages of the two PDs on the *X*-axis, they are the PDs of A and C in Figure 1a. $U_{y_{1}}$ and $U_{y_{2}}$ represent the output voltages of the two PDs on the *Y*-axis, they are the PDs of B and D in Figure 1a.

### 2.2. 2D Deflection Detection Principle

The VCSEL emits a Gauss spot, the intensity of which gradually decreases from the center to the periphery. The spatial distribution of intensity is determined by the following equation:

(4) $$I=\frac{2P_{0}}{\pi R^{2}}\exp\left[\frac{-2\left(x^{2}+y^{2}\right)}{R^{2}}\right]$$

where R is the spot radius while $P_{0}$ is the intensity of the light emitted by VCSEL.

**Figure 3:**
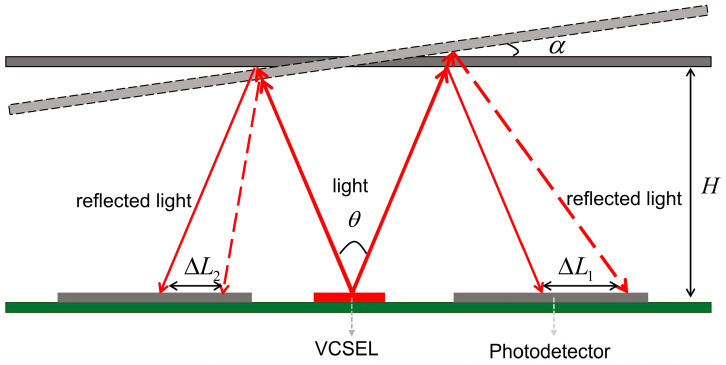
Modeling of the reflected light received by PDs.

The model of the PDs receiving light reflected from the micromirror is shown in Figure 3. In the one-dimensional direction, the two PDs are symmetrical about the VCSEL. When the micromirror is parallel to the receiving surface of the PDs, the reflected spot is in the center of the two PDs, so that the PDs receive the same light intensity. As the micromirror is tilted, the center of the reflected spot shifts, resulting in a corresponding change in the intensity of the received light and the area of the effective spot received by the PDs. The light intensity received by the PDs can be expressed as follows [14]:

(5) $$P=\frac{P_{0}\cos(\alpha )S(x,y,r)}{2\pi H^{2}\tan^{2}\left(\frac{\theta }{2}\right)}\exp\left[\frac{-\left({\left(x+r_{\alpha x}\right)}^{2}+{\left(y+r_{\alpha y}\right)}^{2}\right)}{2H^{2}\tan^{2}\left(\frac{\theta }{2}\right)}\right]$$

where α presents the angle of tilt of the micromirror; H is the vertical distance between the plane of the micromirror and the PD; θ is the beam divergence of the VCSEL, x and y are the coordinates of the PD relative to the VCSEL; r is the distance between the center of VCSEL and the center of the PD; S is the effective spot area received by the PD; $r_{\alpha x}$ and $r_{\alpha y}$ are the coordinate changes of the spot center on the *X*-axis and *Y*-axis, respectively, following the tilting of the micromirror. It is essential to ensure that half of the beam divergence of the VCSEL is greater than the maximum tilt angle of the micromirror $\alpha_{\max}$ to prevent the light from being reflected toward only one PD when reaching the maximum angle.

When the micromirror is tilted only about the *X*-axis or *Y*-axis, the displacement distance of the spot of the two PDs, which are perpendicular to the yaw axis [15], are respectively given by the following two equations:

(6) $$\Delta L_{1}=H•\left[\tan\left(\frac{\theta }{2}+\alpha \right)-\tan\frac{\theta }{2}\right]=H•\frac{\tan\alpha }{1+\tan\frac{\theta }{2}•\tan\left(\frac{\theta }{2}+\alpha \right)}$$

(7) $$\Delta L_{2}=H•\left[\tan\frac{\theta }{2}-\tan\left(\frac{\theta }{2}-\alpha \right)\right]=H•\frac{\tan\alpha }{1+\tan\frac{\theta }{2}•\tan\left(\frac{\theta }{2}-\alpha \right)}$$

when θ and α are sufficiently small and the spot boundary does not extend beyond the range of the PDs, the displacement distance of the spot is about H•α. The sum of spot areas on symmetrical PDs is nearly constant. Thus, the area difference ΔS can be expressed by α and H:

(8) ΔS=f(α,H)

The sum-to-difference ratio of light intensity on the symmetric PDs is:

(9) $$\frac{P_{1}-P_{2}}{P_{1}+P_{2}}=\frac{S_{1}-S_{2}}{S_{1}+S_{2}}\approx K•f(\alpha ,H)$$

If the micromirror is then tilted about another axis, the effective spot area received by the two PDs mentioned above changes:

(10) $$\begin{array}{l} {S_{1}}^{\prime }=k_{1}•S_{1}\\ {S_{2}}^{\prime }=k_{2}•S_{2} \end{array}$$

$k_{1}$ and $k_{2}$ are the dynamic coefficients of variation. When $k_{1}$ ≈ $k_{2}$, the sum-to-difference ratio of light intensity still satisfies Equation (9). It can be assumed that the sum-to-difference ratio of the light intensity on the symmetrical PDs varies linearly when H is constant and α is varied uniformly over the angular range of the PD that can be monitored.

**Figure 4:**
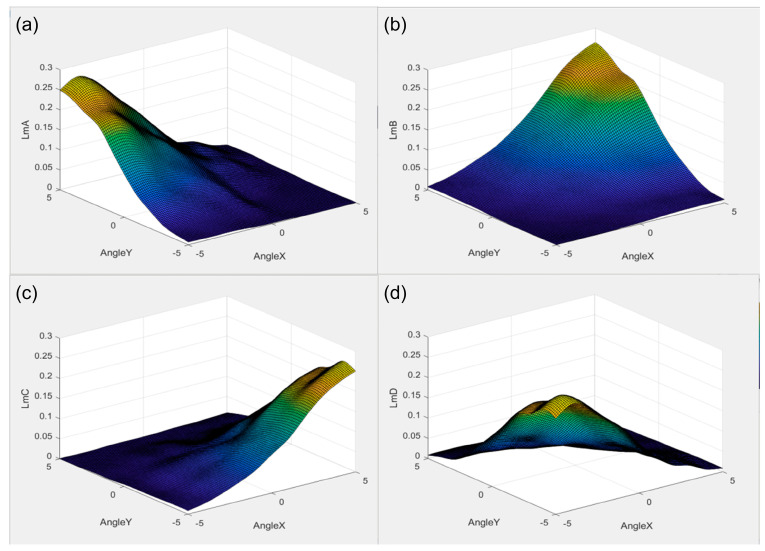
Simulation plot of the value of luminous flux in each quadrant versus the 2D deflection angle of the micromirror: (**a**) PD_A; (**b**) PD_B; (**c**) PD_C; (**d**) PD_D.

The relationship between the luminous flux of the four-quadrant photodetectors and the two-dimensional deflection angle of the micromirror was obtained through the utilization of a Trace Pro simulation. As illustrated in Figure 4, the luminous fluxes of PD_A, PD_B, PD_C, and PD_D reach their maximum values when the micromirror is deflected to the angles [5°, 5°], [5°, 5°], [5°, −5°], and [−5°, −5°], respectively. This information serves as a reference for subsequent experiments.

## 3. Construction of Experimental Platforms and Data Acquisition

### 3.1. Experimental Platform Construction

**Figure 5:**
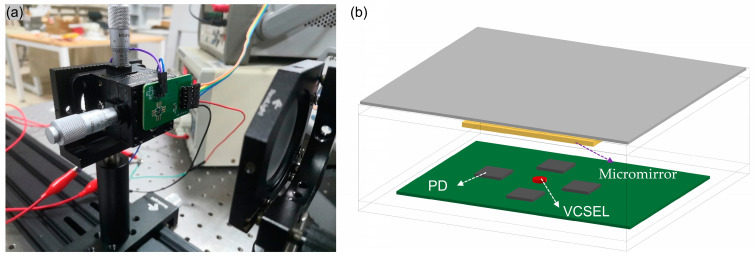
Test platform: (**a**) diagram of the test platform; (**b**) schematic.

The test platform is shown in Figure 5. The construction process follows. The center of the four-quadrant photodetector, where the position of the VCSEL, is aligned with the center of the dual-axis goniometer. At the same time, each side of the four-quadrant photodetector is fixed parallel to the dual-axis goniometer, so that it can rotate around the rotation point of the dual-axis goniometer in order to replace the rotation of the micromirror. However, better reflection is achieved by replacing the micromirrors with larger aluminum mirrors. The dual-axis goniometer with the four-quadrant photodetector and the mirror are mounted on a slide. Adjust the position of both when the outputs of the four PDs are almost equal, bearing in mind that the mirrors should be perfectly parallel to the four-quadrant photodetector.

### 3.2. 2D Deflection Data Acquisition

To avoid the influence of ambient light, the experiments were conducted in a dark room. When the micromirror is tilted only in one direction, the light intensity received by the two symmetrical PDs parallel to the yaw axis is almost the same, while there is a difference in the light intensity received by the two symmetrical PDs perpendicular to the yaw axis. The difference varies with the angle of inclination. The optical flux of the corresponding detector is calculated and processed according to Equation (11):

(11) $$p_{1}=\frac{\Phi_{x_{1}}-\Phi_{x_{2}}}{\Phi_{x_{1}}+\Phi_{x_{2}}},p_{2}=\frac{\Phi_{y_{1}}-\Phi_{y_{2}}}{\Phi_{y_{1}}+\Phi_{y_{2}}}$$

where, $\Phi_{x_{1}}$ and $\Phi_{x_{2}}$ denote the luminous fluxes of the two PDs on the *X*-axis, $\Phi_{y_{1}}$ and $\Phi_{y_{2}}$ denote the luminous fluxes of the two PDs on the *Y*-axis. Thus, the two-dimensional tilting of the micromirror can be decomposed into two one-dimensional tilting models: tilting angle $\alpha_{1}$ around one yaw axis and tilting angle $\alpha_{2}$ around the other yaw axis.

**Figure 6:**
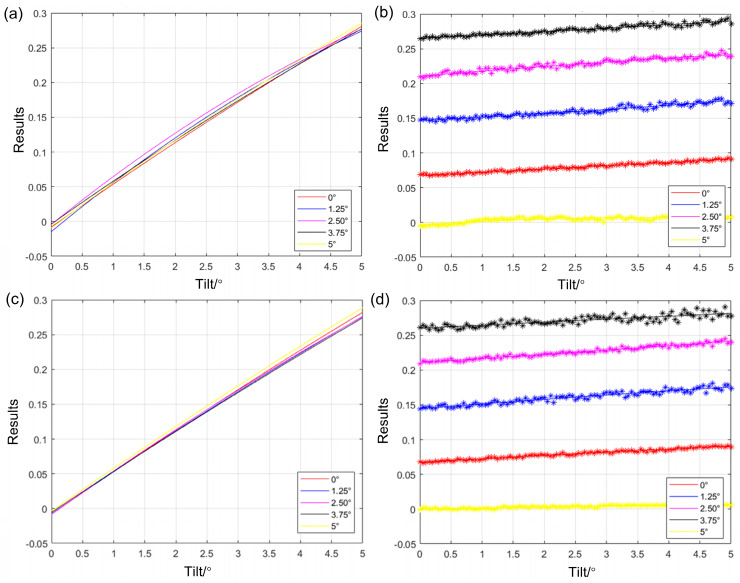
Two-dimensional tilting situation: (**a**) output processing results of PD in the *y*-axis direction when the *x*-axis is the yaw axis; (**b**) output processing results of PD in the *x*-axis direction when the *x*-axis is the yaw axis; (**c**) output processing results of PD in the *x*-axis direction when the *y*-axis is the yaw axis; (**d**) output processing results of PD in the *y*-axis direction when the *y*-axis is the yaw axis.

The experiments were conducted to collect data using the *X*-axis and the *Y*-axis as the yaw axis. The angles of the non-yaw axis were set to 0°, 1.25°, 2.50°, 3.75°, and 5.00°, and the outputs of the four-quadrant photodetector were collected in the range 0 to 5° with 0.05° increments around the yaw axis. When the four-quadrant photodetector is essentially parallel to the mirror, there is still a difference between the two outputs in one direction, so the data point with the smallest difference is set as the 0° data. As illustrated in Figure 6a, the gradients of the fitted curves for the PD output of the non-yaw axis are approximately equal at the five-set angles, indicating that the change in the angle of the non-yaw axis has a negligible effect on the power in the direction near the yaw axis. Furthermore, the power gradient declines linearly with an increase in the non-yaw axis tilt angle. If the total flux of the two PDs in the direction perpendicular to the yaw axis is taken as a constant value, the change in the difference between the two PDs decreases as the tilt angle increases. As Figure 6b illustrates, an increase in the tilt angle of the non-yaw axis results in a gradual increase in the output of the PDs in the yaw axis, although the change is not statistically significant. This indicates that the illuminated area of the two PDs in the direction of the parallel yaw axis is gradually reduced, resulting in a decrease in the denominator of Equation (3). Consequently, the calculated results demonstrate an increasing trend. The resulting image when the *y*-axis is used as the yaw axis is illustrated in Figure 6c,d.

Because the performance of each PD is not identical, and because of random errors inherent in the operation of dual-axis goniometers, there are numerical differences in the tilt when the *Y*-axis and *X*-axis are used as the yaw axis, respectively, but the trends are consistent and correspond well with the theoretical model.

## 4. Regression Prediction Based on the PSO-LSSVM Algorithm

### 4.1. The PSO-LSSVM Algorithm

The objective of the experiment was to calibrate the output of the four-quadrant photodetector concerning the tilt angle. However, due to the insufficient quantity and poor repeatability of the data obtained from multiple experiments, it was impossible to plot an accurate and complete calibration curve solely based on the experimental results. Therefore, it was necessary to apply an algorithm to regressively analyze the data to accurately predict the tilt angle based on the outputs of the four PDs in subsequent measurements.

Suykens. A. K et al. proposed an improved support vector machine method, Least Squares Support Vector Machine (LSSVM) [12], which replaces the inequality constraints in the traditional SVM algorithm with equality constraints. The loss function uses the sum of squares of the errors. This transforms the algorithm into a problem of solving a linear system of equations. The operation process of the LSSVM algorithm is simpler, so the operation time can be greatly reduced. The particle swarm optimization (PSO) was first proposed by Eberhart and Kennedy in 1995 [13]. The core of the algorithm is to simulate individual birds with particles, and the search process of particles is used to simulate the flight process of individual birds. Speed and position, as the only two attributes of the particles, represent the search speed and direction, respectively. According to the historical optimal position of the particles and the population, the flight speed and position of the particles will be continuously transformed in a certain way.

In this paper, the PSO is integrated with the LSSVM to find the optimal kernel function parameters and regularization parameters in LSSVM, to improve the learning ability of the LSSVM and its ability to adapt to new samples. The specific optimization process of the PSO-LSSVM is as follows [16].

(1) Initialization of model parameters. The initial values of the parameters are set according to the complexity of the model, the required convergence speed, and other factors. The initialized position and speed of each particle are set randomly in the search space and speed interval.
(2) Identify and locate extreme values and optimal solutions in the space under consideration. The specific problem under consideration defines an appropriate fitness function. The regression error is employed as the function’s return value in order to ascertain its fitness parameter, which is then subjected to a minimum value optimization search. The minimum value of the fitness parameter identified during the search process is defined as the individual extreme value and recorded in accordance with its corresponding position. The minimum value among the individual extreme values of all particles is defined as the current global optimal solution. If the current global optimum is better than the historical global optimum, then it is referred to as the new global optimum, and its location is recorded.
(3) Update the particle position and velocity. In each iteration, the velocity and position of the particle are transformed according to the acceleration factor and the optimal position, as shown in Equations (12) and (13) [17].
  (12) $$v_{id}^{k+1}=v_{id}^{k}+c_{1}r_{1}^{k}(p_{id}^{k}-x_{id}^{k})+c_{2}r_{2}^{k}(p_{gd}^{k}-x_{gd}^{k})$$
  (13) $$x_{id}^{k+1}=x_{id}^{k}+v_{id}^{k+1}d=1,2,\cdot \cdot \cdot ,D$$
  where $v_{id}^{k}$ and $x_{id}^{k}$ are the velocity and position of the $i_{th}$ particle in d dimensional parameter at the $k_{th}$ iteration; $\gamma_{1}^{k}$ and $\gamma_{2}^{k}$ are two random numbers in the range of [0, 1]; $c_{1}$ and $c_{2}$ are acceleration factors; $p_{id}^{k}$ and $p_{gd}^{k}$ are the individual and global optimal positions of the $i_{th}$ particle in the d dimensional parameter, respectively.
(4) Algorithm termination. When the number of iterations reaches the set maximum (maxgen) or the global optimal solution (the regression error) meets the expected requirements, the optimized LSSVM parameters are output and used in the subsequent LSSVM algorithms.

**Figure 7:**
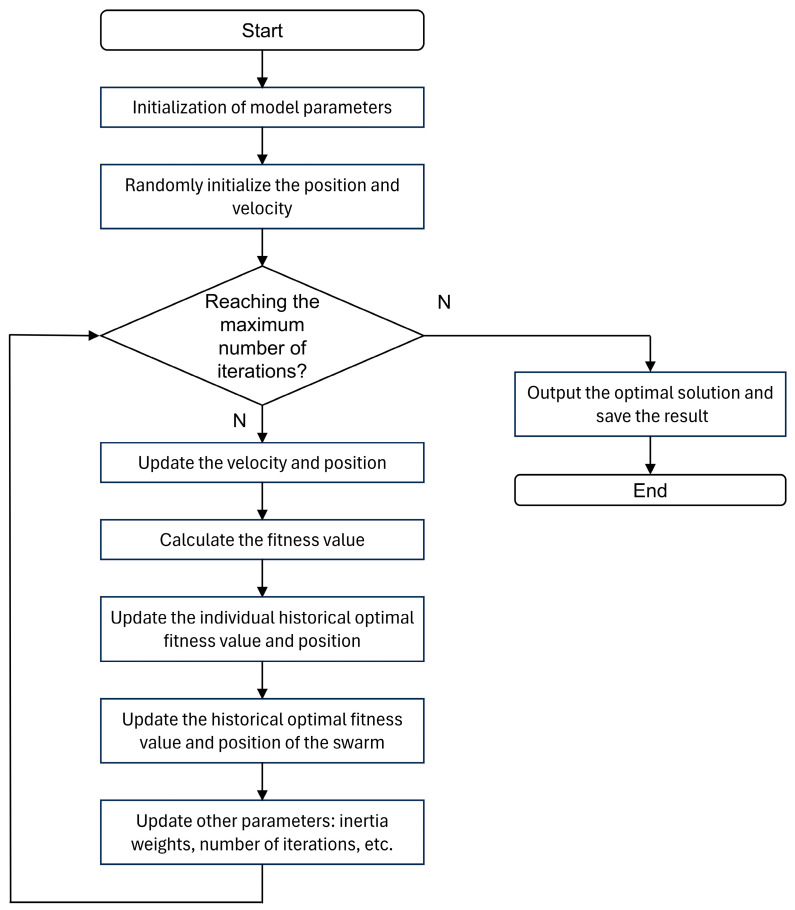
Flowchart of PSO-LSSVM algorithm.

The specific optimization flow of the PSO-LSSVM algorithm is shown in Figure 7.

### 4.2. Regression Prediction Based on PSO-LSSVM

**Figure 8:**
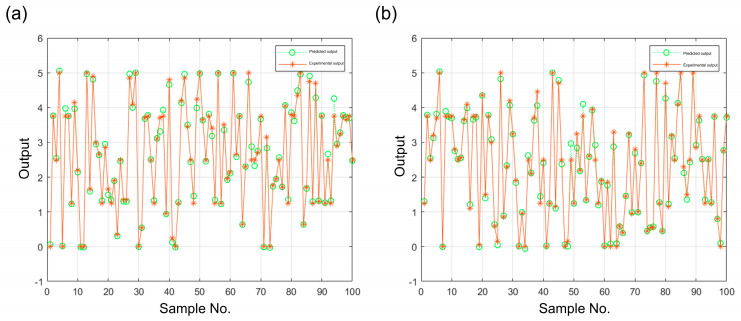
Test prediction effects: (**a**) around the *x*-axis; (**b**) around the *y*-axis.

A PSO-LSSVM was employed to generate a regression model using the results of the PD output in the directions of the *x*-axis and *y*-axis as inputs and the actual rotation angles around the *x*-axis and *y*-axis as outputs. A total of 900 groups of training samples and 100 groups of test data were set. The regression effects of the tilt angle around the *x*-axis and *y*-axis are illustrated in Figure 8. The green dots represent the output predicted by the algorithm, and the orange dots depict the experimental actual production. Upon observation, it can be seen that there is a notable degree of similarity between the algorithm output and the experimental output in these 100 samples. The regression effect of the two-dimensional angular deflection model is evaluated using three error indicators: mean absolute error (MAE), decision factor ($R^{2}$), and mean square error (MSE).

The mean absolute error (MAE) is calculated as,

(14) $$MAE=\frac{1}{N}\sum_{i=1}^{N}\left|y_{i}-{y_{i}}^{*}\right|$$

The mean square error (MSE) is calculated as,

(15) $$MSE=\frac{1}{N}\sum_{i=1}^{N}{\left(y_{i}-{y_{i}}^{*}\right)}^{2}$$

The decision factor ($R^{2}$) is calculated as,

(16) $$R^{2}=1-\frac{\sum_{i=1}^{N}{\left(y_{i}-{y_{i}}^{*}\right)}^{2}}{\sum_{i=1}^{N}{\left(y_{i}-\bar{y}\right)}^{2}}$$

In the following, take the deflection angle of the micromirror along the *x*-axis as an example, as shown in Table 1.

A reduction in MAE and MSE with the $R^{2}$ approaching 1 are indicative of enhanced predictive capability on the part of the model. The deflection angle of the micromirror was accurately predicted based on the voltage of the PDs. However, since the measured data are affected by systematic and random errors, there is more room for improvement in the MAE and MSE. By further adjusting the acceleration constants and weighting coefficients, the accuracy and speed of the regression prediction can be improved and eventually applied to the micromirror angle monitored system.

## 5. Conclusions

In this paper, the PSO-LSSVM algorithm was employed for the purpose of performing a retrospective analysis of the data of the micromirror’s two-dimensional deflection. The coefficients of determination of the fitted models around the *X*-axis and the *Y*-axis were 0.9947 and 0.9913, respectively, with mean-square errors of approximately 0.0091° and 0.0072°. A feasible method is proposed for the calibration of micromirrors and their measurement systems, which enables the prediction of micromirror tilt angles. The result may be employed as an input for actuator control, functioning as a feedback signal to facilitate the correction of the motion state of the MEMS micromirror, thereby enabling the attainment of heightened precision through closed-loop control.

## Figures and Tables

**Table 1:** PSO-LSSVM model assessment metrics (Unit: °).

	MAE	MSE	R^2^
Around the *x*-axis	0.06566849	0.00908366	0.9947
Around the *y*-axis	0.05283867	0.00715366	0.9913

## Data Availability

Data are contained within the article.
